# Role of Orai3-Mediated Store-Operated Calcium Entry in Radiation-Induced Brain Microvascular Endothelial Cell Injury

**DOI:** 10.3390/ijms24076818

**Published:** 2023-04-06

**Authors:** Qibing Wu, Yang Fang, Xiaoyu Huang, Fan Zheng, Shaobo Ma, Xinchen Zhang, Tingting Han, Huiwen Gao, Bing Shen

**Affiliations:** 1 Department of Radiotherapy, The First Affiliated Hospital of Anhui Medical University, Hefei 230032, China; 2 School of Basic Medical Sciences, Anhui Medical University, Hefei 230032, China

**Keywords:** store-operated Ca^2+^ channels, Orai3, X-ray, brain microvascular endothelial cell, radiotherapy, brain injury

## Abstract

Radiation-induced brain injury is a serious complication with complex pathogenesis that may accompany radiotherapy of head and neck tumors. Although studies have shown that calcium (Ca^2+^) signaling may be involved in the occurrence and development of radiation-induced brain injury, the underlying molecular mechanisms are not well understood. In this study, we used real-time quantitative polymerase chain reaction and Western blotting assays to verify our previous finding using next-generation sequencing that the mRNA and protein expression levels of Orai3 in rat brain microvascular endothelial cells (rBMECs) increased after X-ray irradiation. We next explored the role of Orai3 and Orai3-mediated store-operated Ca^2+^ entry (SOCE) in radiation-induced brain injury. Primary cultured rBMECs derived from wild-type and Orai3 knockout (Orai3^(−/−)^) Sprague–Dawley rats were used for in vitro experiments. Orai3-mediated SOCE was significantly increased in rBMECs after X-ray irradiation. However, X-ray irradiation-induced SOCE increase was markedly reduced in Orai3 knockout rBMECs, and the percentage of BTP2 (a nonselective inhibitor of Orai channels)-inhibited SOCE was significantly decreased in Orai3 knockout rBMECs. Functional studies indicated that X-ray irradiation decreased rBMEC proliferation, migration, and tube formation (a model for assessing angiogenesis) but increased rBMEC apoptosis, all of which were ameliorated by BTP2. In addition, occurrences of all four functional deficits were suppressed in X-ray irradiation-exposed rBMECs derived from Orai3^(−/−)^ rats. Cerebrovascular damage caused by whole-brain X-ray irradiation was much less in Orai3^(−/−)^ rats than in wild-type rats. These findings provide evidence that Orai3-mediated SOCE plays an important role in radiation-induced rBMEC damage and brain injury and suggest that Orai3 may warrant development as a potential therapeutic target for reducing or preventing radiation-induced brain injury.

## 1. Introduction

Radiotherapy with X-rays, γ-rays, and other ionizing radiation is a common method for the treatment of local malignant tumor lesions [1]. For head and neck tumors, radiotherapy acts directly on the tumor lesions to obtain better clinical effects. However, at the same time, the normal tissues around the lesion may also be damaged by radiation, which may lead to blood–brain barrier (BBB) damage and brain edema [1]. Radiation-induced brain injury may appear in patients during or after radiotherapy [2] and can cause brain damage, cognitive decline, and long-term mental health damage to patients, all of which have serious negative impacts on patient survival and quality of life [3]. Because radiation-induced brain injury involves complex processes, the mechanisms of its occurrence remain unclear. Exploring the mechanisms of the occurrence and development of radiation-induced brain injury and seeking to reduce brain injury caused by radiation therapy are important for resolving this limitation of radiation therapy.

In recent years, studies have reported that microvascular damage is a key factor in the occurrence of radiation-induced brain damage in the central nervous system [4]. Cerebral microvascular endothelial cells are a key component of brain microvessels and the BBB. Most studies investigating the effects of X-ray irradiation on vascular injury have focused on vascular endothelial cells because they are highly sensitive to radiation [5,6,7]. Andrews et al. showed that radiation causes changes in vascular endothelial cell apoptosis, damages the integrity of endothelial tight junctions, and decreases microvessel density, thereby increasing the permeability of blood vessels, leading to the destruction of the BBB and the occurrence of brain injury [4].

Our team recently used next-generation sequencing to explore changes in the transcription profile of rat brain microvascular endothelial cells (rBMECs) after radiation exposure [8]. We found that calcium (Ca^2+^) signals including 38 genes were significantly changed. Ca^2+^ is an important ion involved in signal transduction in cells, which extensively regulates cell functions. Any changes in intracellular Ca^2+^ concentration [Ca^2+^]_i_ affect almost all endothelial cell functions [9,10]. Studies have found that exposure to ionizing radiation causes a large influx of extracellular Ca^2+^ into cells, leading to Ca^2+^ overload and cell dysfunction [11,12]. Store-operated Ca^2+^ entry (SOCE) is an important pathway for mediating Ca^2+^ influx in non-excitable cells. SOCE is regulated by the Ca^2+^ sensor stromal interacting molecule, which is located in the endoplasmic reticulum [9]. The Orai channel is an important Ca^2+^ channel that mediates Ca^2+^ influx through SOCE. Proteins in the Orai family, including Orai1, Orai2, and Orai3, form highly selective Ca^2+^ channels on the plasma membrane [13]. In our previous study, we analyzed the transcript changes in rBMECs after X-ray irradiation and found that the transcript expressions of Oria1, Orai2, and Orai3 were all significantly increased, but Orai3 was the most significantly changed of the three genes [8]. As an important SOCE component, Orai3 is closely related to the function of vascular smooth muscle cells and endothelial cells under pathophysiological conditions [14,15]. Here, we hypothesized that changes in the expression of Orai3 would be involved in the process of brain damage caused by radiation. Therefore, in this study, we used rats with wild-type (WT) Orai3 (Orai3^(+/+)^) and rats with Orai3 knockout (Orai3^(−/−)^) to study the role and mechanisms whereby Orai3 may be involved in radiation-induced damage of BMCEs. Understanding these underlying processes may reveal new targets for the development of clinical radiation-induced brain injury prevention and treatment.

## 2. Results

### 2.1. X-ray Irradiation Increases Orai3 Expression in rBMECs

**Figure 1:**
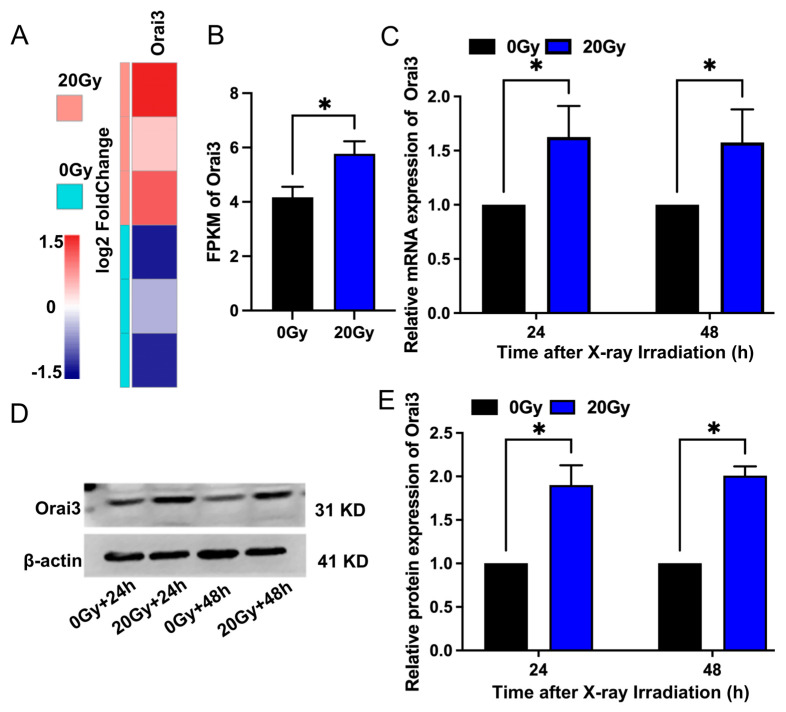
Effect of X-ray irradiation on Orai3 expression levels in rat brain microvascular endothelial cells (rBMECs). (**A**) Orai3 transcript-based heatmap. Different color intensities represent expression levels, with blue and red representing low and high expression levels, respectively. (**B**) Summary data expressed as fragments per kilobase per million (FPKM) mapped reads for Orai3. (**C**) Expression levels of Orai3 mRNA in rBMECs 24 h and 48 h after 20 Gy X-ray irradiation. (**D**,**E**) Representative images (**D**) and summary data (**E**) showing Orai3 protein expression level changes in rBMECs 24 h and 48 h after X-ray irradiation (20 Gy) compared with control (0 Gy). Data are shown as the mean ± SEM (*n* = 3), * *p* < 0.05 analyzed by two-tailed unpaired Student’s *t*-test.

Our previous study using next-generation sequencing found that the transcript expression of Orai3 was significantly increased in rBMECs after X-ray irradiation. Here, we confirmed that X-ray irradiation increased Orai3 transcript expression (Figure 1A,B). In addition, the results of RT-qPCR and Western blotting assays showed that expression levels of both Orai3 mRNA and protein in rBMECs derived from WT rats (rBMECs/WT) were significantly increased 24 h and 48 h after X-ray irradiation (20 Gy) of the cells compared with those in the control group that were not irradiated (Figure 1C–E). These findings, consistent with our previous next-generation sequencing results, indicate that the expression level of Orai3 in rBMECs significantly increases after X-ray irradiation.

### 2.2. Role of Orai3 in X-ray Irradiation-Induced Enhancement of SOCE in rBMECs

**Figure 2:**
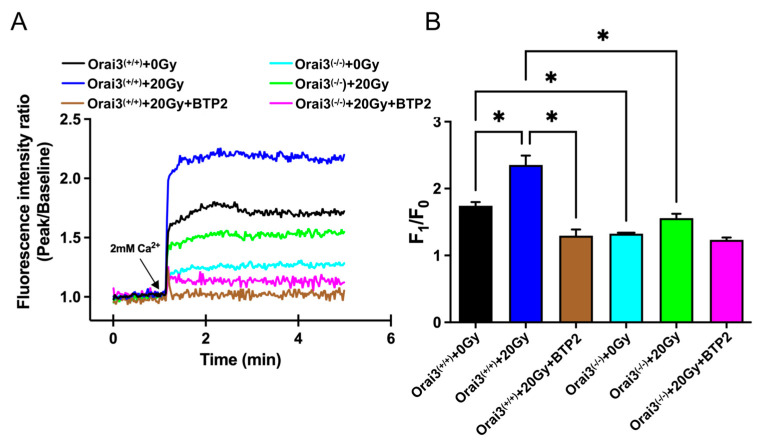
Effect of X-ray irradiation on store-operated Ca^2+^ entry (SOCE) in rat brain microvascular endothelial cells (rBMECs). (**A**,**B**) Representative traces (**A**) and summary data (**B**) showing thapsigargin (TG) -evoked SOCE in rBMECs derived from wild-type Orai3^(+/+)^ rats 24 h after cells were unexposed (0 Gy) or exposed (20 Gy) to X-ray irradiation and untreated or treated with BTP2 (5 μM); and in rBMECs derived from rats with Orai3 knockout (Orai3^(−/−)^)unexposed (0 Gy) or exposed to X-ray irradiation (20 Gy) and untreated or treated with BTP2 (5 μM). After cellular Ca^2+^ stores were depleted by the application of 2 μM TG for 10 min in Ca^2+^-free medium, SOCE was evoked by application of 2 mM Ca^2+^ to the cell culture medium. Data are shown as the mean ± SEM (*n* = 3), * *p* < 0.05 analyzed by one-way analysis of variance followed by Dunnett’s multiple comparisons test.

An important function of Orai3 is to mediate the Ca^2+^ influx by SOCE. We speculated that the increased expression of Orai3 in rBMECs caused by X-ray irradiation would enhance the SOCE mediated by Orai3, triggering a large Ca^2+^ influx and inducing a Ca^2+^ overload to disrupt Ca^2+^ homeostasis in these cells. To test this hypothesis, we conducted Ca^2+^ imaging experiments to explore the changes in [Ca^2+^]_i_ of rBMECs. After Ca^2+^ stores were depleted by thapsigargin (2 μM) in a Ca^2+^-free solution, SOCE was evoked by adding 2 mM Ca^2+^ into bath solution. Our Ca^2+^ imaging results showed that compared with that in control rBMECs derived from WT rats (rBMECs/WT-0Gy), SOCE-mediated Ca^2+^ influx was significantly increased in X-ray irradiated rBMECs derived from WT rats (rBMECs/WT-20Gy) (Figure 2A,B). The enhanced Ca^2+^ influx caused by X-ray irradiation in rBMECs/WT was significantly inhibited by the addition of N-(4-[3,5-bis(trifluoromethyl)-1H-pyrazol-1-yl]phenyl)-4-methyl-1,2,3-thiadiazole-5-carboxamide (BTP2), a nonselective inhibitor of Orai channels (Figure 2A,B). In rBMECs derived from Orai3^(−/−)^ rats (rBMECs/KO-0Gy), the SOCE-mediated [Ca^2+^]_i_ rise was significantly reduced compared with rBMECs/WT-0Gy, but significantly enhanced by X-ray irradiation (rBMECs/KO-20Gy) compared with that in rBMECs from Orai3^(-/-)^ control group (rBMECs/KO-0Gy) (Figure 2A,B). However, SOCE in rBMECs/KO-20Gy group was dramatically reduced compared with rBMECs/WT-20Gy. Although BTP2 still significantly suppressed SOCE in rBMECs/KO-20Gy, the percentage of X-ray irradiation-enhanced SOCE was largely reduced in rBMECs/KO (61 ± 11 vs. 23 ± 5, *p* < 0.05) (Figure 2A,B). Together, these results suggest that the increase in SOCE caused by X-ray irradiation may be partially mediated by Orai3.

### 2.3. Role of Orai3 in X-ray Irradiation-Induced Inhibition of rBMEC Proliferation

**Figure 3:**
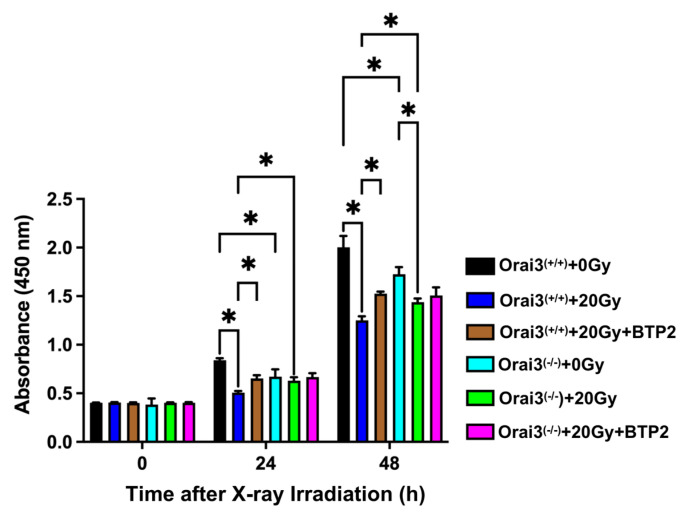
Role of Orai3 in X-ray irradiation-induced inhibition in the proliferation of rat brain microvascular endothelial cells (rBMECs). Summary data showing rBMEC proliferation as assessed using CCK8 assays 0 h, 24 h, and 48 h after rBMECs derived from wild-type rats (Orai3^(+/+)^) that were unexposed (0 Gy) or exposed (20 Gy) to X-ray irradiation and untreated or treated with BTP2 (5 μM); and rBMEC proliferation after cells from Orai3 knockout rats (Orai3^(−/−)^) were unexposed (0 Gy) or exposed to X-ray irradiation (20 Gy) and treated or untreated with BTP2 (5 μM). Data are shown as the mean ± SEM (*n* = 3), * *p* < 0.05 analyzed by two-way analysis of variance followed by Dunnett’s multiple comparisons test.

Because changes in [Ca^2+^]_i_ will affect various biological processes, including cell proliferation, migration, and apoptosis, we next explored the role of Orai3 in the functional changes of rBMECs after X-ray irradiation. We used CCK8 assays to detect rBMEC proliferation. Compared with rBMECs/WT-0Gy, the proliferation of rBMECs/WT-20Gy was significantly inhibited both 24 h and 48 h after exposure, and this inhibition was stronger at 48 h (Figure 3). This inhibitory effect was significantly reduced by the application of BTP2, indicating that the inhibition of rBMEC proliferation may be related to SOCE (Figure 3). In addition, compared with rBMECs/WT-0Gy, the proliferation was significantly reduced in rBMECs/KO-0Gy. Compared with rBMECs/WT-20Gy, X-ray irradiation-induced inhibition of rBMEC proliferation was significantly ameliorated in rBMECs/WT with BTP2 treatment and rBMECs/KO without BTP2 treatment, even X-ray irradiation also inhibited the proliferation of rBMECs/KO only in 48 h group (Figure 3). More importantly, BTP2 application to these rBMECs/KO did not alter the degree of the ameliorated inhibition of proliferation (Figure 3). These results indicate that Orai3-mediated SOCE may be involved in X-ray irradiation-induced inhibition of rBMEC proliferation.

### 2.4. Role of Orai3 in X-ray Irradiation-Induced Inhibition of rBMEC Migration

**Figure 4:**
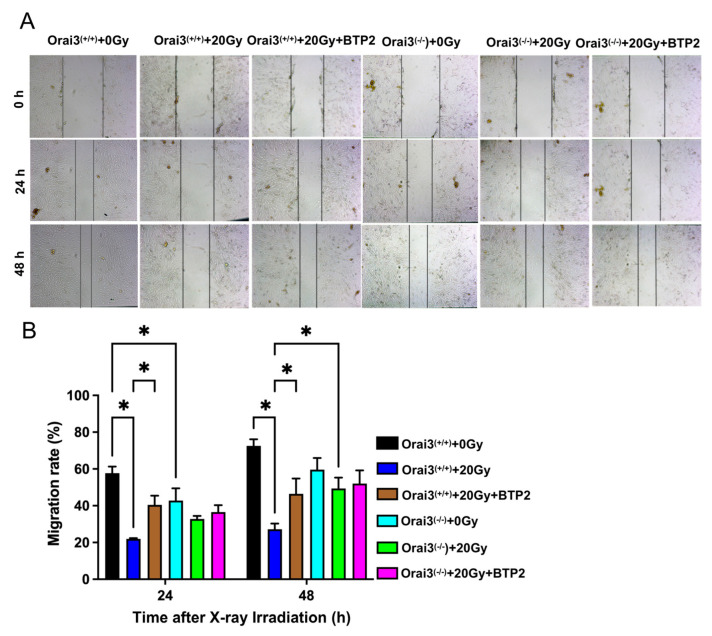
Role of Orai3 in X-ray irradiation-induced inhibition of cell migration in rat brain microvascular endothelial cells (rBMECs). (**A**,**B**) Representative images (**A**) and summary data (**B**) showing cell migration 24 h and 48 h after irradiation for rBMECs derived from wild-type rats (Orai3^(+/+)^) and unexposed (0 Gy) or exposed (20 Gy) to X-ray irradiation and untreated or treated with BTP2 (5 μM); and rBMECs derived from Orai3 knockout rats (Orai3^(−/−)^) unexposed (0 Gy) or exposed to X-ray irradiation (20 Gy) and untreated or treated with BTP2 (5 μM). Data are shown as the mean ± SEM (*n* = 3), * *p* < 0.05 analyzed by two-way analysis of variance followed by Dunnett’s multiple comparisons test.

We used a wound healing assay, also called a cell scratch assay, to detect cell migration in each experimental group. The scratches in the cell fields were photographed at 0 h, 24 h, and 48 h after X-ray irradiation. In 24 h group, our results showed that, compared with rBMECs/WT-0Gy, the migration rates of rBMECs/WT-20Gy and rBMECs/KO were significantly decreased, and BTP2 significantly ameliorated X-ray irradiation-induced inhibition of the migration in rBMECs/WT (Figure 4). In addition, X-ray irradiation did not significantly affect the migration in rBMECs/KO, and BTP2 did not have an effect on the migration in rBMECs/KO-20Gy (Figure 4).

In 48 h group, the migration rate for rBMECs/WT was also markedly inhibited by X-ray irradiation, and both BTP2 treatment and Orai3 knockout significantly ameliorated the migration inhibited by X-ray irradiation (Figure 4). In addition, BTP2 did not have an effect on the migration in rBMECs/KO-20Gy (Figure 4). Together, these findings indicate that Orai3-mediated SOCE may participate in the inhibition of rBMEC migration caused by X-ray irradiation and that either pharmacological inhibition or knockout of Orai3 may alleviate X-ray irradiation-induced inhibition of this migration.

### 2.5. Role of Orai3 in X-ray Irradiation-Induced Inhibition of rBMEC Tube Formation

**Figure 5:**
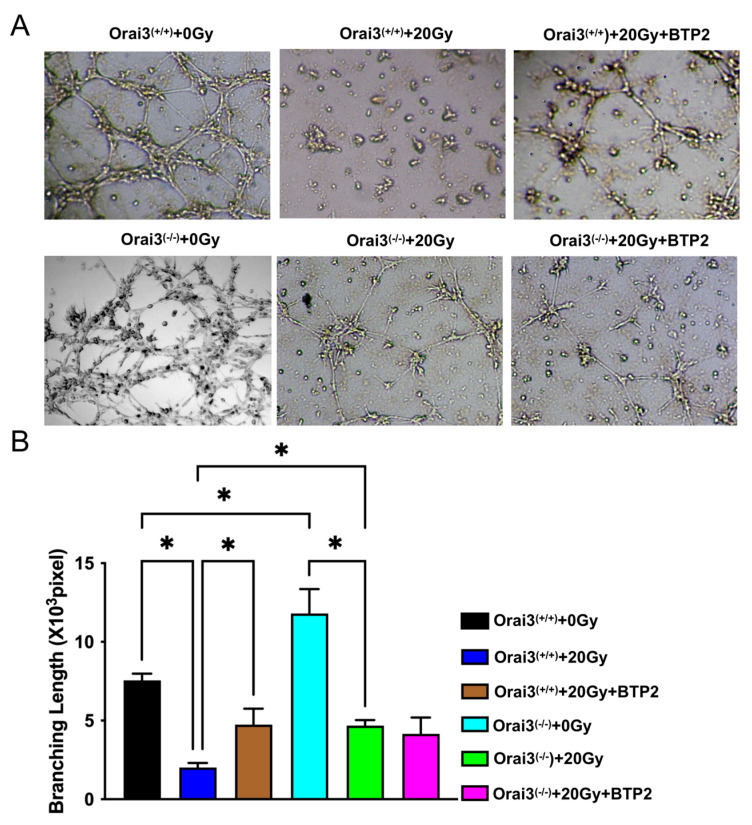
Role of Orai3 in X-ray irradiation-induced inhibition of tube formation by rat brain microvascular endothelial cells (rBMECs). (**A**,**B**) Representative images of formed tubes (**A**) and summary data (**B**) of tube formation 24 h after rBMECs derived from wild-type in Orai3^(+/+)^ were unexposed (0 Gy) or exposed (20 Gy) to X-ray irradiation and untreated or treated with BTP2 (5 μM); and rBMECs derived from Orai3 knockout rats (Orai3^(−/−)^) were unexposed (0 Gy) or exposed to X-ray irradiation (20 Gy) and untreated or treated with BTP2 (5 μM). Data are shown as the mean ± SEM (*n* = 3), * *p* < 0.05 analyzed by one-way analysis of variance followed by Dunnett’s multiple comparisons test.

The ability of vascular endothelial cells to proliferate and migrate is closely related to their ability to form tubes. To explore the role of Orai3 in tube formation by rBMCEs, we used a Matrigel experiment. The rBMECs were unexposed or exposed to X-ray irradiation (20 Gy), and tube formation was evaluated after 24 h. Our data showed that, compared with rBMECs/WT-0Gy, the lengths of the tubes formed by rBMECs were significantly shorter in rBMECs/WT-20Gy. This finding indicated that X-ray irradiation may inhibit rBMEC angiogenesis (Figure 5A,B). This decreased tube formation was partially ameliorated by the application of BTP2 (Figure 5A,B). Compared with rBMECs/WT-0Gy, the tube formation was significantly increased in rBMECs/KO-0Gy but X-ray irradiation also strongly suppressed the tube formation in rBMECs/KO (Figure 5A,B). Thus, this increased tube formation in rBMECs/KO may be caused by Orai3 knockout-induced increment in the tube formation. More importantly, BTP2 did not have ameliorated effect on X-ray irradiation-inhibited tube formation (Figure 5A,B). These findings indicate that SOCE may be involved in X-ray irradiation-induced inhibition of rBMEC tube formation. They further suggest that inhibiting SOCE may alleviate the X-ray irradiation-induced inhibition of rBMEC tube formation. Radiation caused inhibition of tube formation in rBMECs/WT as well as rBMECs/KO. Therefore, radiation-induced effects on tube formation are independent of presence of Orai3 as tube formation is inhibited in both rBMECs/WT and rBMECs/KO. Moreover, BTP2 only partially rescued the inhibition in rBMECs/WT and did not rescue at all in rBMECs/KO. Tube formation was increased after knocking down Orai3.

### 2.6. Role of Orai3 in X-ray Irradiation-Induced Apoptosis of rBMECs

**Figure 6:**
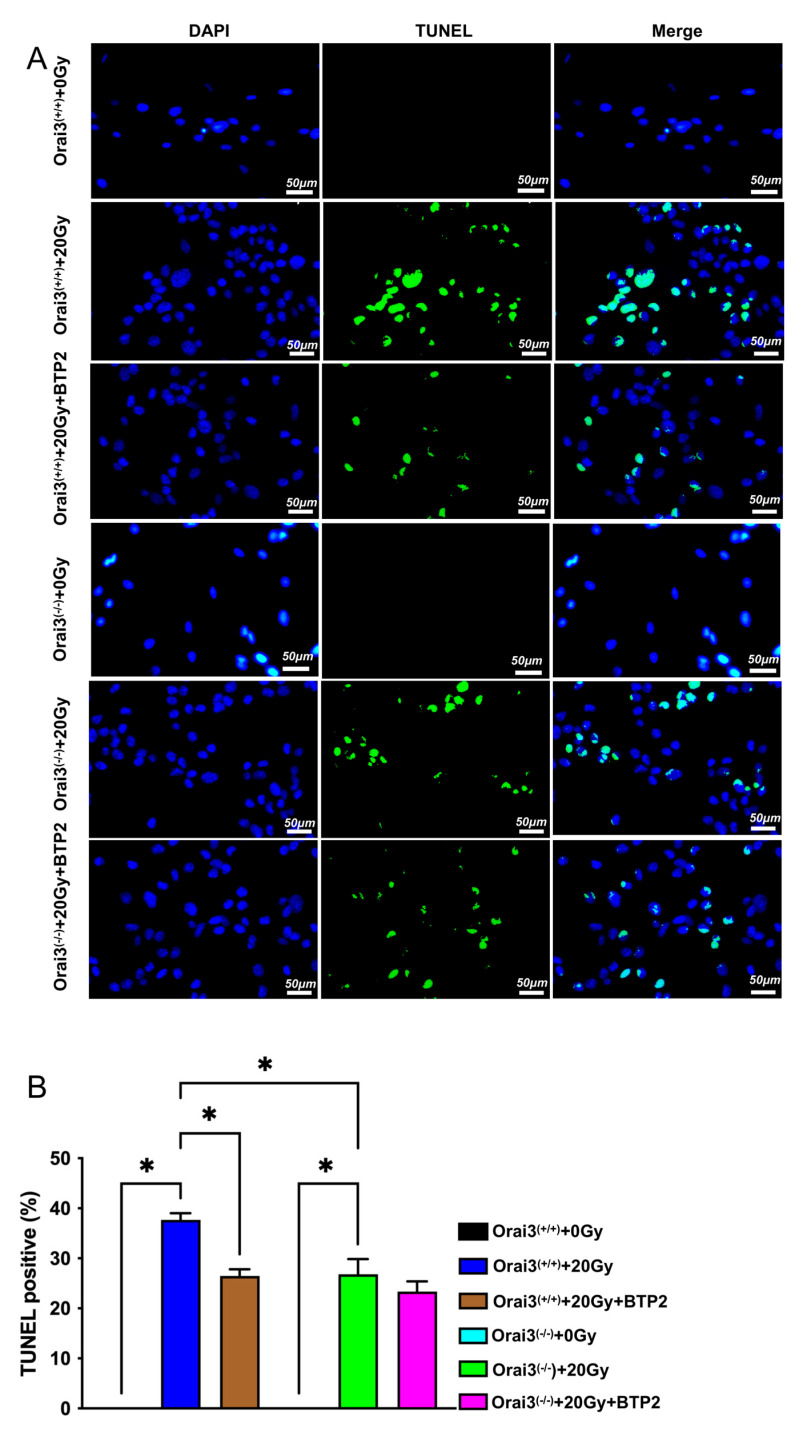
Role of Orai3 in X-ray irradiation-induced apoptosis of rat brain microvascular endothelial cells (rBMECs). (**A**,**B**) Representative images of TUNEL staining (**A**) and summary data (**B**) showing rBMEC apoptosis 24 h after cells derived from wild-type rats (Orai3^(+/+)^) and unexposed (0 Gy) or exposed to X-ray irradiation (20 Gy) and untreated or treated with BTP2 (5 μM); and cells derived from Orai3 knockout rats (Orai3^(−/−)^) and unexposed (0 Gy) or exposed to X-ray irradiation and untreated or treated with BTP2 (5 μM). Green represents apoptotic cells stained through the TUNEL assay and blue indicates the cell nucleus stained by 4’,6-diamidino-2-phenylindole. Data are shown as the mean ± SEM (*n* = 3), * *p* < 0.05 analyzed by one-way analysis of variance followed by Dunnett’s multiple comparisons test.

Many studies have shown that X-ray irradiation may induce cell apoptosis [16]. To explore the role of Orai3 in X-ray irradiation-induced apoptosis, we used TUNEL assays to detect rBMEC apoptosis. Our results showed that, compared with rBMECs/WT-0Gy and rBMECs/KO-0Gy, respectively, the rate of apoptosis significantly increased after exposure to 20 Gy X-ray irradiation, indicating that X-ray irradiation induced the apoptosis of rBMEC both from WT and Orai3^(−/−)^ rats (Figure 6A,B). BTP2 treatment in rBMECs/WT and rBMECs/KO significantly inhibited X-ray irradiation-induced apoptosis (Figure 6A,B). More importantly, BTP2 did not have ameliorated effect on X-ray irradiation-induced apoptosis and even X-ray irradiation also significantly enhanced the apoptosis in rBMECs/KO (Figure 6A,B). These findings suggest that Orai3-mediated SOCE participates in radiation-induced apoptosis of rBMECs and that inhibition of Orai3-mediated SOCE or knockout of Orai3 may reduce this apoptosis.

**Figure 7:**
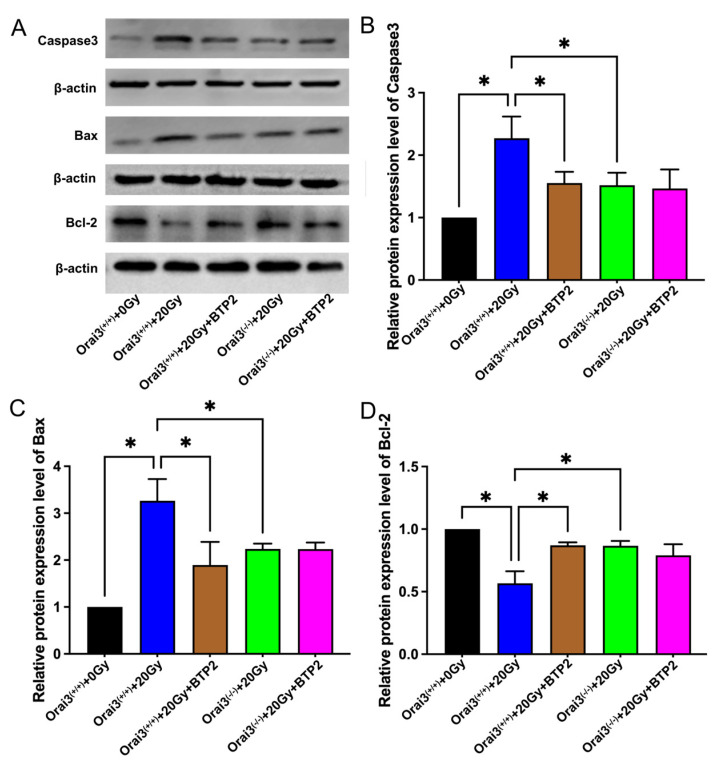
Role of Orai3 in X-ray irradiation-induced changes in expression levels of apoptosis-related proteins in rat brain microvascular endothelial cells (rBMECs). (**A**–**D**) Representative images (**A**) and summary data (**B**–**D**) showing caspase3, Bax, and Bcl-2 protein expression levels in rBMECs derived from wild-type (Orai3^(+/+)^) rats unexposed (0 Gy) or exposed to X-ray irradiation (20 Gy) and untreated or treated with BTP2 (5 μM); and from Orai3 knockout rats (Orai3^(+/+)^) exposed to X-ray irradiation (20 Gy) and untreated or treated with BTP2 (5 μM). Data are shown as the mean ± SEM (*n* = 3), * *p* < 0.05 analyzed by one-way analysis of variance followed by Dunnett’s multiple comparisons test.

We also used Western blotting to detect the expression of the apoptosis-related proteins Bax and caspase3 and the apoptosis antagonist protein Bcl-2 in rBMECs. Compared with those in rBMECs/WT, the expression levels of Bax and caspase3 in rBMECs/WT-20Gy were significantly increased, whereas the expression of Bcl-2 was significantly decreased (Figure 7A–D). These alterations were significantly suppressed by the application of BTP2. Compared with those in rBMECs/WT-20Gy, the expression levels of Bax and caspase3 in rBMECs/KO-20Gy were significantly decreased, whereas the expression of Bcl-2 was significantly increased (Figure 7A–D); application of BTP2 did not further alter the expression of any of these proteins (Figure 7A–D). These findings support those obtained using TUNEL assays and suggest that inhibition of Orai3 and Orai3-mediated SOCE may reduce radiation-induced rBMEC apoptosis.

### 2.7. Role of Orai3 in X-ray Irradiation-Induced Rat Brain Tissue Injury

**Figure 8:**
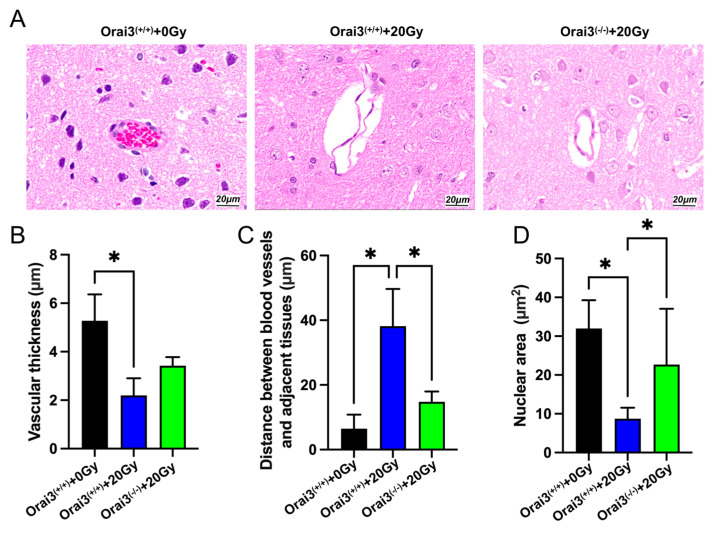
Role of Orai3 in X-ray irradiation-induced rat brain tissue injury. Brain tissue histopathological sections showing tissue changes in wild-type rats (Orai3^(+/+)^) after whole-brain exposure to a single dose of X-ray irradiation (20 Gy), and Orai3 knockout rats (Orai3^(-/-)^) exposed to the same radiation. (**A**) Representative images showing hematoxylin and eosin (HE) staining of brain tissue slices. (**B**-**D**) Quantification of vascular thickness (**B**, *n* = 3), distance between blood vessels and surrounding tissues (**C**, *n* = 3), and nuclear area of vascular cells (**D**, *n* = 9) in brain tissue slices. Data are shown as the mean ± SEM, * *p* < 0.05 analyzed by one-way analysis of variance followed by Dunnett’s multiple comparisons test.

Previous studies using a single dose of X-rays (2, 6, 8, 19.5, 22, 30, or 50 Gy) to irradiate Sprague–Dawley rats established a rat model of radiation-induced injury and detected an increase in vascular permeability 24 h after irradiation [17,18]. To further explore the role of Orai3 in radiation-induced brain vascular injury, we used an X-ray irradiation dose of 20 Gy to expose the whole brain of WT and Orai3^(−/−)^ rats and performed hematoxylin and eosin staining to observe the brain vascular damage (Figure 8A). Brain tissue slices showed that cells in the walls of blood vessels in the brains of WT rats unexposed to radiation were intact with no obvious pathological changes. By contrast, cells in blood vessel walls in the brains of irradiated rats were smaller and deformed and displayed nuclear pyknosis, and the gaps between the blood vessels and surrounding tissues were enlarged (Figure 8B,D). In comparison, the degree of nuclear pyknosis and of the enlargement in the perivascular space of Orai3^(−/−)^ rats that had been exposed to the same level of radiation were reduced (Figure 8C,D). These results indicate that the knockout of Orai3 may reduce the cerebrovascular damage caused by X-ray irradiation.

## 3. Discussion

The mechanisms undergirding radiation-induced brain injury are not fully understood. Many studies have reported that ionizing radiation may induce endothelial cell deformation, apoptosis, tight junction impairment, vasodilation, permeability increase, and other pathological changes, leading to BBB destruction and brain injury [19]. Our previous study found that the changes in the transcript expression profile of rBMECs after X-ray irradiation are related to Ca^2+^ signaling pathways [8]. In the present study, we confirmed the results of our previous study that the expression of Orai3 in rBMECs is significantly increased after X-ray irradiation. The Orai channel family includes three homologues: Orai1, Orai2, and Orai3. As a member of the Orai protein family, Orai3 mediates SOCE to allow for Ca^2+^ influx. Thus, we also assessed and confirmed our previous finding that X-ray irradiation increases Orai3-mediated SOCE [20]. Application of a nonselective inhibitor of Orai channels, BTP2, to rBMECs derived from Orai3 knockout rats and exposed to radiation showed that X-ray irradiation-induced SOCE increase was markedly reduced in rBMECs/KO and the percentage of BTP2-inhibited SOCE was largely decreased in rBMECs/KO, indicating that it was mainly mediated by Orai3. Besides Orai3, there should be some other pathways including Orai1, Orai2, and other channels to involve in this X-ray irradiation-induced SOCE because X-ray irradiation still enhanced SOCE slightly in rBMECs/KO. SOCE changes caused by abnormalities in Orai3 have been closely related to a variety of diseases. For example, one study has shown that Orai3 is the SOCE-mediated protein that plays a major role in estrogen receptor-positive breast cancer cells [21]. Increased expression of Orai3 in cardiomyocytes has been associated with the occurrence of cardiomyocyte hypertrophy [22]. In addition, increased expression of Orai3 may indicate a poor prognosis in colon cancer [6]. However, to date, there are no reports on the role of Orai3 in radiation-induced brain injury.

Ca^2+^ is an important second messenger in endothelial cells that regulates various functions of these cells, including proliferation, migration, angiogenesis, and apoptosis [23,24]. Several studies have shown that ionizing radiation can inhibit the proliferation of cerebrovascular endothelial cells, enhance apoptosis, aging, and calcium signaling of cerebrovascular endothelial cells, and ultimately lead to the destruction of the BBB, thereby promoting the occurrence of radiation-induced brain injury [20,25,26,27]. To explore the role of Orai3 in X-ray irradiation-induced damage of rBMECs, WT and Orai3^(−/−)^ rats were used in the present study to conduct cell and animal experiments. We found that, after rBMECs/WT were irradiated with X-rays at a dose of 20 Gy, cell migration and proliferation and tube formation, a model for assessing angiogenesis, were significantly inhibited, and cell apoptosis was significantly increased. In rBMECs/WT and treated with the nonselective Orai inhibitor BTP2 and in rBMECs/KO, inhibition of cell migration and proliferation caused by X-ray irradiation were alleviated, and cell apoptosis was reduced. The tube formation was enhanced in rBMECs/KO but only alleviated in BTP2 treatment in rBMECs/WT irradiated by X-ray. These results indicated that inhibition of Orai3-mediated SOCE may reduce X-ray irradiation-induced functional damage in rBMECs.

Radiotherapy of the central nervous system causes acute apoptosis of endothelial cells, leading to endothelial damage and acute destruction of the BBB [18]. In the animal experiments conducted in the present study, we compared WT and Orai3^(−/−)^ rats to find that whole-brain X-ray irradiation induced fewer cerebrovascular tissue lesions in Orai3^(−/−)^ rats. This finding shows that Orai3 may be involved in the occurrence and development of X-ray irradiation-induced brain injury and that knockout of Orai3 may have a protective effect against X-ray irradiation-induced cerebrovascular damage. For clinical prevention or treatment of X-ray irradiation-induced brain injury, there is currently a lack of target-specific drugs with curative effects and low adverse effects. Therefore, exploring the mechanisms underlying vascular endothelial cell radiation injury and finding possible therapeutic targets has recently become an important direction in radiological research [28,29]. The present study found that inhibiting or knocking out the Orai3 Ca^2+^ channel alleviated the functional damage to rBMECs caused by X-ray irradiation. The mechanism for the decreased functional damage may involve the inhibition of Ca^2+^ influx to reduce X-ray irradiation-induced intracellular Ca^2+^ overload. Therefore, the present study may inform the understanding of the mechanisms underpinning X-ray irradiation brain injury and provides a new potential target for the prevention and treatment of clinical radiation-induced brain injury. However, we have to face to a possible side effect for inhibition of Orai3. In our study, we found that SOCE is defected in rBMECs/KO, which may affect some physiological functions of rBMECs. In a future study, we will evaluate and provide further comments.

## 4. Materials and Methods

### 4.1. Animals and Cells

We obtained Orai3^(−/−)^ Sprague–Dawley rats from the Nanjing Institute of Biomedicine, Nanjing University [30]. Orai3^(−/−)^ and Orai3^(+/+)^ WT littermates (200 ± 20 g) were raised indoors, and all rats used in experiments were male. All animal experiments were conducted according to the National Institutes of Health guidelines (Publication No. 8523) and were approved by the Animal Experimentation Ethics Committee of Anhui Medical University. The rats were kept in an animal room with a constant temperature of 23 ± 1 °C and allowed free access to food and water.

The cells used in this experiment were primary cultures of rBMECs, and the process used to obtain these cells has been described previously [8]. Briefly, the rats were humanely killed by asphyxiation with CO_2_, and the cerebral cortex tissues were collected and cut into pieces with a volume of 1 mm^3^. The tissue was digested with 0.2% type II collagenase solution (C2-BIOC, Sigma, Saint Louis, MO, USA). Medium containing 10% fetal bovine serum (Cat. No. 10099, Gibco, Grand Island, NY, USA) and 1% phosphate-buffered saline (PBS; Cat. No. 10010031, Gibco, USA) was used for cell culture medium, and cells were cultured at 37 °C in 5% CO_2_. The medium was replaced with fresh medium after 24 h.

### 4.2. X-ray Irradiation

X-ray irradiation was conducted similarly to our previous study [8]. When the density of the primary cultured rBMECs was approximately 70%, a linear accelerator, model CLINIC600C (Varian, Palo Alto, CA, USA), was used to irradiate the cells with a single X-ray dose of 20 Gy at a distance of 100 cm with an irradiation field of 10 cm × 10 cm. The dose rate was 250C Gy/min. A water film with a thickness of 1.0 cm was placed on the cell culture dish to ensure the consistency of the dose across the field. The control group used sham irradiation, with no X-ray exposure but all other procedures were the same as those for the cells in the irradiated group.

Three WT rats and three Orai3^(−/−)^ rats weighing 200 ± 20 g were subjected to a single X-ray irradiation of the whole brain at a dose of 20 Gy. For the control group, the dose was 0 Gy. The rats were anesthetized with an intraperitoneal injection of ketamine (50 mg/kg) and xylazine (10 mg/kg). A CLINIC600C linear accelerator was used to irradiate the whole brain of the rats at a dose rate of 250C Gy/min, a source-to-skin distance of 100 cm, and an irradiation field size of 3 cm × 3 cm.

### 4.3. Reverse-Transcription Quantitative Real-Time Polymerase Chain Reaction (RT-qPCR) Assay

Total RNA Extraction Reagent (Cat. No. R401-01, Vazyme, Nanjing, China) was used to isolate total RNA from rBMECs, and a PrimeScript RT Master Mix kit (Cat. No. Q111-02, Vazyme, Nanjing, China) was used for reverse transcription. We used the SYBR Green I fluorescence method for quantification. The mRNA levels were normalized to β-actin. The forward primer sequence for β-actin was 5′-CCCATCTATGAGGGTTACGC-3′, and the reverse primer sequence was 5′-TTTAATGTCACGCACGATTTC-3′. The forward primer sequence for Orai3 was 5′-CCCATATTGAAGCCGTGAGC-3′, and reverse primer sequence was 5′-TCCACATATCGGTGGAGTCG-3′. The 2^−ΔΔCT^ method was used to calculate the relative gene expression levels of the control groups and the groups exposed to X-ray irradiation.

### 4.4. Western Blotting

Radioimmunoprecipitation assay lysis buffer (Cat. No. P0013B, Beyotime, Shanghai, China) was used to lyse rBMECs, and the bicinchoninic acid assay method is used to quantify the levels of protein. Sodium dodecyl sulfate–polyacrylamide gel electrophoresis (10% gels) was used to separate equal amounts of proteins. Proteins were transferred to polyvinylidene difluoride membranes, which were subsequently blocked with 5% fat-free milk in PBS containing 0.1% Tween 20 at room temperature for 1 h. The membranes were incubated with the following primary antibodies overnight at 4 °C: Orai3 (Cat. No. SAB3500447, Sigma, dilution ratio 1:1000), Bcl-2 (Cat. No. 16026-1-AP, Proteintech, San Diego, CA, USA, dilution ratio 1:1000), Bax (Cat. No. 60267-1-Ig, Proteintech, dilution ratio 1:1000), caspase3 (Cat. No. 66470-2-Ig, Proteintech, dilution ratio 1:1000), or β-actin (Cat. No. sc-47778, Santa Cruz Biotechnology, Dallas, TX, USA, dilution ratio 1:1000). The next day, secondary goat IgG antibody (Cat. Nos. S0002 or S0010, Affinity Biosciences, Melbourne, Australia, dilution ratio 1:5000) was incubated with the membranes for 1 h. Chemiluminescence gel imaging was used to analyze the immunoblot by densitometry.

### 4.5. Wound Healing Assay

The rBMECs were inoculated onto a 12-well plate, with the same cell density in each well. When the cells became 80% confluent, they were scratched (“wounded”) with a sterile pipette tip. After one rinse with PBS, the medium was changed to be serum-free and the cells were cultured in a 37 °C, 5% CO_2_ incubator. Images of the cells in the wells were obtained at 0 h, 24 h, and 48 h after radiation exposure. ImageJ software was used to analyze the area of traces and to calculate the cell migration rate. The cell migration rate was calculated using the formula: [(0 h scratch area − 24 h or 48 h scratch area)/0 h scratch area] × 100%.

### 4.6. Cell Viability Assay

The rBMECs were evenly seeded in 96-well plates at a density of 5000 cells per well. At three time points (0 h, 24 h, and 48 h), we discarded the medium, washed the cells twice with PBS, and then incubated the cells with 10 μL of CCK8 (K1018, Apexbio, Houston, TX, USA) in each well. After placing the 96-well plate in the incubator for another 2 h, we detected the absorbance (optical density) at a wavelength of 450 nm using a microplate reader (Caretium, Shenzhen, China).

### 4.7. Tube Formation Assay

The ability of the rBMECs to form tubes (a model of angiogenesis) was assessed using a Matrigel tube formation assay. A 96-well plate was placed on ice, and a pre-cooled pipette tip was used to add 50 µL of Matrigel (E1270, Corning, Corning, NY, USA) to each well. The plate was placed in a 37 °C cell incubator for 1 h. A suspension of rBMECs (200 µL) was added to the Matrigel-coated wells. The plates were then incubated at 37 °C for 8 h. An inverted microscope (Nikon, Tokyo, Japan) was used to obtain images of the cells.

### 4.8. TUNEL Assay

The amount of rBMCE apoptosis was evaluated after X-ray irradiation by using a Vazyme TUNEL BrightGreen Apoptosis Detection kit (Cat. No. A112-01; Nanjing, China). Briefly, the cells were seeded in a 48-well plate and were subjected to irradiation once cell density reached 70% confluency. Twenty-four hours after irradiation, the apoptosis detection experiment was carried out. The cells were fixed with 4% paraformaldehyde and permeabilized with 0.3% Triton X-100. A solution of 1× Equilibration Buffer was used to cover the cells, and TdT incubation buffer was added to each well. The cells were incubated for 1 h at 37 °C. A counterstain of 4’,6-diamidino-2-phenylindole (2 μg/mL) was added dropwise at room temperature and protected from light for 5 min. After the cells were washed with 1× PBS, the fluorescence signal was captured using a fluorescence microscope.

### 4.9. [Ca^2+^]_i_ Measurement

[Ca^2+^]_i_ measurements were performed as previously described [31,32]. The rBMCEs were uniformly seeded in 96-well plates and subjected to X-ray irradiation or other treatments. After 24 h, the medium was replaced with 100 μL of medium containing Fluo-8 solution (Invitrogen, Waltham, MA, USA), and the cells were incubated at 37 °C in 5% CO_2_ for 30 min. The Ca^2+^ stores in the rBMCEs were depleted by the addition of thapsigargin (TG; 2 μM, Sigma, USA) in a Ca^2+^-free solution (OPSS, 140 mM NaCl, 5 mM KCl, 2 mM CaCl_2_, 1 mM MgCl_2_, 10 mM glucose, and 5 mM HEPES, pH 7.3 to 7.4 adjusted with NaOH). Then, 2 mM CaCl_2_ was added to the cell medium by using the automanipulator of a FlexStation 3 system. We added N-(4-[3,5-bis(trifluoromethyl)-1H-pyrazol-1-yl]phenyl)-4-methyl-1,2,3-thiadiazole-5-carboxamide (BTP2; 5 μM; Cat. No. HY-100831, MedChemExpress, Monmouth Junction, NJ, USA) to some wells to block the Orai channels. Data were expressed as a fluorescence intensity ratio: peak/baseline (peak is the maximum value of fluorescence intensity after 2 mM Ca^2+^ addition, and baseline is the average of baseline value of fluorescence intensity for 30 s prior to Ca^2+^ addition). The ratio is calculated as the peak value divided by the baseline value.

### 4.10. Hematoxylin and Eosin Staining Assay

Rats were anesthetized by an intraperitoneal injection of ketamine (50 mg/kg) and xylazine (10 mg/kg). The brain was quickly removed after cardiac perfusion, fixed with 4% paraformaldehyde (Servicebio, Wuhan, China) for 24 h, sectioned, and stained using a standard protocol with hematoxylin and eosin.

### 4.11. Statistical Analysis

All data are expressed as mean ± SEM. GraphPad Prism software (version 8.3.0.538, San Diego, CA, USA) was used for statistical analysis and graphing. One-way and two-way analysis of variance, or a *t*-test to compare two groups, was used to evaluate the data, and a *p* value < 0.05 was considered statistically significant.

## 5. Conclusions

In summary, our study demonstrated that X-ray irradiation enhanced the expression levels of Orai3 and Orai3-mediated SOCE in rBMECs and that inhibition or knockout of Orai3 may alleviate X-ray irradiation-induced inhibition of cell migration and proliferation and ameliorate rBMEC apoptosis, but may be not directly involved in alleviating X-ray irradiation-induced inhibition of tube formation. Knockout of Orai3 may reduce X-ray irradiation-induced vascular damage. These findings provide new insights into the mechanisms of X-ray irradiation-induced brain injury and offer a potential target for therapeutic drug development.

## Data Availability

Data will be available by the corresponding author under reasonable request.
